# My Health in My Hands: Improving Medication Abortion Knowledge and Closing Disparities with a Community-Led Media Intervention

**DOI:** 10.1089/heq.2023.0210

**Published:** 2024-02-29

**Authors:** Hayley V. McMahon, Leslie Serrano, Teja Vyavahare, Indya Hairston, Sequoia Ayala, Zainab Jah, Tiffany Hailstorks, Dázon D. Diallo, Elizabeth A. Mosley

**Affiliations:** ^1^ Department of Behavioral, Social, and Health Education Sciences, Rollins School of Public Health, Emory University, Atlanta, Georgia, USA.; ^2^ The Center for Reproductive Health Research in the Southeast (RISE), Rollins School of Public Health, Emory University, Atlanta, Georgia, USA.; ^3^ SisterLove, Inc., Atlanta, Georgia, USA.; ^4^ The Raben Group, Washington, District of Columbia, USA.; ^5^ National Birth Equity Collaborative, New Orleans, Louisiana, USA.; ^6^ Department of Gynecology and Obstetrics, Emory University School of Medicine, Atlanta, Georgia, USA.; ^7^ Center for Innovative Research on Gender Health Equity, University of Pittsburgh School of Medicine, Pittsburgh, Pennsylvania, USA.

**Keywords:** medication abortion, reproductive justice, community-led research, community-based participatory research, health literacy

## Abstract

**Purpose:**

Inaccurate beliefs about medication abortion (MA) are common. This study evaluated pilot data from a community-led media intervention designed to increase MA knowledge among Black and Latinx women in Georgia.

**Methods:**

Participants (*N*=855) viewed the intervention video and completed pre–post surveys. Data were analyzed using linear and logistic regression.

**Results:**

Knowledge scores significantly increased from 3.88/5.00 to 4.47/5.00. Participants who were Native American, Asian and Pacific Islander, multiracial, Black, <20 years old, and living in Georgia scored below the sample mean at baseline; however, nearly all disparities disappeared after intervention exposure.

**Conclusions:**

This intervention effectively increased MA knowledge and narrowed racial/ethnic, age-based, and geographic disparities.

## Introduction

Most abortions in the United States are medication abortions (MAs).^1^ With at least 20 states having now enacted abortion bans after the *Dobbs v. Jackson Women's Health Organization* ruling, use of abortion pills has only become more common as access to in-clinic abortion care wanes.^2,3^ Nevertheless, awareness about MA remains relatively low, and inaccurate beliefs are common.^4^ The impacts of this are likely greater in Black and Latinx communities, which experience poorer health literacy, higher unintended pregnancy rates, and more barriers to abortion care—each driven by systemic racism.^5–9^ Despite these racism-based disparities being well established, MA research that centers Black and Latinx populations remains sparse.^10^

The reproductive justice (RJ) framework, a paradigm shift cultivated by Black feminists to center marginalized peoples, points to accessible health information as essential for self-determination.^11^ RJ consists of three core pillars of bodily autonomy: (1) “the right *not* to have a child,” (2) “the right to *have* a child,” and (3) “the right to *parent* children in safe and healthy environments.”^12^ As such, SisterLove, a community-based and Black-led RJ organization based in Atlanta, Georgia, created and led the Georgia Medication Abortion Project (GAMA).

GAMA is a community-led RJ-grounded project designed to assess and increase Black and Latinx Georgians' MA knowledge and access.^10^ Formative research with Black and Latinx women living in Georgia identified a stakeholder desire for community-generated educational media.^13^ Given this, this study aimed to evaluate pilot data from a new GAMA media intervention designed to increase MA knowledge in Black and Latinx communities.

## Materials and Methods

“My Health in My Hands” is a 3-min animated video featuring Damaris, a SisterLove health educator, sharing information about MA processes, safety, effectiveness, and legality with a diverse circle of community members. Qualitative interviews with Black and Latinx women living in Georgia and lived experiences of the SisterLove team were used to identify priority elements of MA and inform the cultural tailoring of materials.^13^

Participants completed the pretest survey, viewed the video, and completed the post-test survey in immediate succession on their own devices. Participants were recruited through Facebook and Twitter and self-selected into the study. Because we sought to identify potential disparities and compare intervention effectiveness by sociodemographic characteristics, being under the age of 18 years was the only exclusion criterion. Participants were compensated with a $15 gift card. All study activities were approved by the institutional review board at Emory University (IRB 00107733).

The primary outcome was MA knowledge scores. The pre- and post-test surveys consisted of identical multiple-choice questions. Each of the five knowledge items was scored as correct (1) or incorrect (0). Knowledge scores ranged from 0 to 5, with 5 indicating all answers were correct. The pretest also included four demographic items on race/ethnicity, age, gender, and geographic location. The post-test included one item on MA awareness: “Prior to the video, did you know about medication abortion?” Linear and logistic regressions were used to assess (1) the mean difference in knowledge scores, (2) the mean difference in knowledge scores by demographic characteristic, and (3) the odds of answering each item correctly. PROC GENMOD in SAS 9.4 was used for analysis.^14^

## Results

Our sample (*N*=855) was diverse in terms of age, geographic location, and race/ethnicity (Table 1). The overall mean pretest score was 3.88 (Table 2). White ($\bar{x}$=4.06) and Latinx ($\bar{x}$=4.40) participants' pretest scores were above the mean, whereas Native American ($\bar{x}$=2.84), Asian and Pacific Islander ($\bar{x}$=2.85), multiracial ($\bar{x}$=2.84), and Black ($\bar{x}$=3.84) participants' scores fell below the mean. Regarding age and geographic location, participants who were <20 years old ($\bar{x}$=3.08) and living in Georgia ($\bar{x}$=3.42) scored significantly lower than the mean.

**Table 1.** Sample Characteristics (*N*=855)

Demographic characteristic	% (***n***)
Geographic location	
Georgia	13.6 (116)
United States, not Georgia	82.5 (705)
Outside of United States	3.9 (33)
Gender	
Woman	83.9 (717)
Man	15.2 (130)
Nonbinary, gender nonconforming	0.7 (6)
Age (years)	
Under 20	4.4 (38)
20–24	22.7 (192)
25–30	31.0 (265)
30–34	27.6 (236)
Over 35	14.4 (123)
Race/ethnicity	
Black, African American, AfroCaribbean	61.8 (528)
Latina/o, Latinx, Latin American, Hispanic	2.3 (20)
Asian, Pacific Islander, Native Hawaiian	1.6 (14)
Native American, Indigenous	2.2 (19)
White, Caucasian	25.7 (220)
Multiracial	1.1 (9)

**Table 2.** Mean Pretest Score, Mean Post-Test Score, and Mean Difference, by Demographic Group (*N*=855)

Demographic group	Pretest score, mean (95% CI)	Post-test score, mean (95% CI)	Difference, mean (95% CI)	***p***
Overall	3.88 (3.79 to 3.96)	4.47 (4.42 to 4.52)	0.59 (0.51 to 0.66)	<0.0001
Geographic location				
Georgia	3.42 (3.18 to 3.66)	4.30 (4.18 to 4.43)	0.88 (0.63 to 1.13)	<0.0001
United States, outside of Georgia	3.95 (3.86 to 4.04)	4.50 (4.45 to 4.56)	0.55 (0.47 to 0.64)	<0.0001
Outside of United States	4.00 (3.65 to 4.35)	4.27 (3.92 to 4.62)	0.27 (−0.02 to 0.57)	0.0693
Gender				
Woman	3.94 (3.33 to 4.03)	4.52 (4.48 to 4.57)	0.58 (0.50 to 0.66)	<0.0001
Nonbinary, gender nonconforming	3.50 (2.61 to 4.39)	4.33 (3.95 to 4.71)	0.83 (0.12 to 1.55)	0.0229
Man	3.54 (3.33 to 3.75)	4.16 (3.98 to 4.34)	0.62 (0.41 to 0.83)	<0.0001
Age (years)				
Under 20	3.08 (2.68 to 3.48)	4.39 (4.19 to 4.60)	1.32 (0.85 to 1.78)	<0.0001
20–24	3.77 (3.60 to 3.94)	4.61 (4.51 to 4.71)	0.84 (0.68 to 1.00)	<0.0001
25–29	3.83 (3.69 to 3.98)	4.42 (4.34 to 4.50)	0.59 (0.45 to 0.73)	<0.0001
30–34	4.16 (4.02 to 4.30)	4.56 (4.47 to 4.64)	0.39 (0.27 to 0.52)	<0.0001
35 or older	3.68 (3.64 to 4.08)	4.20 (4.03 to 4.37)	0.34 (0.14 to 0.54)	<0.0001
Race/ethnicity				
Black, African American	3.84 (3.74 to 3.94)	4.50 (4.45 to 4.56)	0.66 (0.57 to 0.76)	<0.0001
Latinx, Latina/o Hispanic	4.40 (3.95 to 4.85)	4.85 (4.64 to 5.10)	0.46 (0.07 to 0.83)	0.0199
Asian, Pacific Islander	2.85 (2.14 to 3.55)	3.61 (2.99 to 4.24)	0.77 (0.24 to 1.30)	0.0044
Native American, Indigenous	2.84 (2.29 to 3.39)	4.63 (4.37 to 4.89)	1.79 (1.08 to 2.50)	<0.0001
White, Caucasian	4.06 (3.91 to 4.22)	4.37 (4.27 to 4.47)	0.30 (0.16 to 0.45)	<0.0001
Multiracial	3.56 (3.11 to 4.00)	4.56 (4.23 to 4.88)	1.00 (0.56 to 1.44)	<0.0001

CI, confidence interval.

After participants' exposure to the intervention, the post-test sample mean increased by 0.59 to 4.47 (Table 2). The odds of answering correctly also increased for all items (Table 3). There was a significant improvement in knowledge scores by race/ethnicity (χ^2^=25.74, *p*=0.001), age (χ^2^=30.13, *p*≤0.0001), and geographic location (χ^2^=9.38, *p*=0.0092). Gender was not a significant predictor of change in knowledge (χ^2^=0.56, *p*=0.7567).

**Table 3.** Proportion of Participants Answering Correctly and Odds of Answering Correctly in Pretest and Post-Test, by Survey Item (*N*=855)

Survey item	Proportion correct, pretest, %	Proportion correct, post-test, %	Odds ratio (95% CI)	***p***
1. Medication abortion uses_____instead of surgery to end a pregnancy.	77.19	94.62	5.20 (3.71 to 7.27)	<0.0001
2. The medicine used for medication abortions works_____% of the time up to 11 weeks of pregnancy.	64.68	73.10	1.48 (1.20 to 1.83)	0.0002
3. In the state of Georgia, law requires that a patient must wait at least_____hours between scheduling an appointment and receiving medication abortion pills.	78.36	90.88	2.75 (2.16 to 3.51)	<0.0001
4. Mifepristone and misoprostol are the two medicines that are taken for medication abortion. Mifepristone causes_____.	75.56	91.76	3.60 (2.82 to 4.59)	<0.0001
5. Misoprostol causes side effects such as bleeding and cramping to complete the abortion—true or false?	93.10	97.20	2.57 (1.63 to 4.05)	<0.0001

Participants who were Native American (*d*=1.79, *p*<0.0001), multiracial (*d*=1.00, *p*<0.0001), Asian and Pacific Islander (*d*=0.77, *p*=0.0044), Black (*d*=0.66, *p*<0.0001), <20 years old (*d*=1.32, *p*<0.0001), and living in Georgia (*d*=0.88, *p*<0.0001) experienced the greatest increases. In total, 73.1% of participants reported having heard of MA before viewing the video.

## Discussion

This study is one of the first to center Black and Latinx communities in MA research, as well as one of the few reproductive health interventions to utilize a community-led and RJ-grounded design. Overall, the intervention appeared to be effective. Mean differences in pre–post scores were statistically significant for all groups except people living outside the United States (*p*=0.0693), a population that unsurprisingly requires tailoring to their specific context. Significant increases in the odds of correctly answering each survey item also demonstrate that the intervention was associated with immediate improvements in knowledge for all MA elements that were identified as priorities through formative research (Table 3).^13^

Notably, we identified substantial disparities in MA knowledge at baseline. Participants who were Native American, Asian and Pacific Islander, multiracial, Black, <20 years old, and living in Georgia had knowledge scores below the sample mean. However, nearly all of these disparities disappeared after the intervention. Only the disparity observed among Asian and Pacific Islander participants remained. A sizable improvement in knowledge scores (*d*=0.77, *p*=0.0044) was achieved in this group, but MA research that is culturally tailored to this specific population is likely needed.^15^ In contrast, Latinx participants had the highest mean scores, which is somewhat surprising given the existing literature on health literacy disparities.^7,16^

Further qualitative research is needed, but it may be that the decades-long history of self-managed MA in many Latin American countries has driven greater MA knowledge in Latinx communities.^17^ Interestingly, Native American participants had the greatest increase in knowledge (*d*=1.79, *p*<0.0001), despite the lack of formative research with this population. Although this intervention was designed for Black and Latinx audiences, it appears to be well suited to Native American audiences as well.

This pilot study has several notable limitations, including a self-selected nonrepresentative sample and short-term measurement through a relatively small number of items. We also did not collect participants' IP addresses for privacy reasons; therefore, we cannot rule out the possibility that our data may contain duplicate responses. Furthermore, only 2.3% (*n*=20) of our sample population was Latinx; therefore, our ability to draw conclusions related to this population is limited.

The forthcoming implementation of this intervention will include survey refinement by incorporating additional knowledge items and a validated scale on abortion attitudes, strengthening the recruitment strategy, and updating information on legality as state policies continue to shift. Future research should investigate the longitudinal retention of knowledge and additional strategies for culturally tailoring MA knowledge interventions, particularly for Asian and Pacific Islander populations.

## Conclusions

With a wave of abortion bans and associated misinformation rolling across much of the country after the demise of *Roe v. Wade*, it is more important than ever that advocates and health educators have evidence-based tools to promote accurate information about MA.^18^ This is particularly important for Black and Latinx communities, which concurrently have both the greatest need for and poorest access to abortion care.^8^ Concerningly, our baseline findings documented racial/ethnic, age-based, and geographic disparities in MA knowledge.

However, this analysis also demonstrated that a culturally tailored media intervention can effectively increase MA knowledge in diverse audiences and largely eliminate existing disparities. Given these results, we believe that this intervention could be successfully adapted for other populations through community-engaged formative research and ultimately serve as a resource in the work toward RJ for all.

## Data Availability

Survey data are not publicly available. However, the latest version of the My Health in My Hands video is available for use by community-based organizations and health educators. Find it in English at (bit.ly/MyHealthInMyHands-English) and in Spanish at (bit.ly/MyHealthInMyHands-Spanish).

## Abbreviations Used

CI: confidence interval
GAMA: Georgia Medication Abortion Project
MA: medication abortion
RJ: reproductive justice
